# Autophagy and the Mitochondrial Lon1 Protease Are Necessary for *Botrytis cinerea* Heat Adaptation

**DOI:** 10.1111/mmi.70014

**Published:** 2025-07-18

**Authors:** Mingzhe Zhang, Liang Ma, Zhiqun Lyu, Naomi Kagan Trushina, Amir Sharon

**Affiliations:** ^1^ School of Plant Sciences and Food Security Tel Aviv University Tel Aviv Israel; ^2^ National‐Regional Joint Engineering Research Center for Soil Pollution Control and Remediation in South China, Guangdong Key Laboratory of Integrated Agro‐Environmental Pollution Control and Management, Institute of Eco‐Environmental and Soil Sciences Guangdong Academy of Sciences Guangzhou P.R. China

## Abstract

Heat adaptation is a multilayered universal process involving a coordinated response of general and heat‐specific cellular systems and processes. Here, we demonstrate that adaptation of the plant pathogenic fungus *Botrytis cinerea* to mild heat stress requires both autophagy and the mitochondrial Lon1 protease. Deleting *bclon1* or blocking autophagy by deleting the *bcatg1* autophagy‐regulating gene did not affect fungal survival at optimal temperature. Under heat stress, deletion of *bclon1* induced earlier and more intense autophagy, mitochondrial malfunction, and accelerated fungal cell death. These phenomena were intensified in a *bcatg1/lon1* double mutant, indicating coordinated activity of both pathways in heat adaptation. Blocking autophagy, but not *bclon1*, also affected mycelia growth, spore germination, as well as nuclei division and spore morphology. Our results support a cytoprotective role for autophagy downstream of mitochondria‐driven death signals, possibly as a mechanism that promotes growth arrest and helps remove damaged cellular components.

## Graphical abstract

The destabilization of mitochondria due to the disruption of the Lon1 protease induces cellular damage, including reactive oxygen species (ROS) accumulation, impaired germination, and growth arrest. Activation of autophagy inhibits fungal growth and recycles damaged cellular components, potentially serving as a cytoprotective role downstream of the mitochondria‐driven death signal.

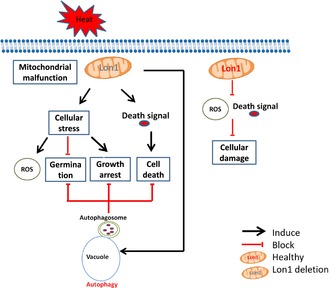

## 1 Introduction

*Botrytis cinerea* is a common fungal plant pathogen with a necrotrophic lifestyle that causes gray mold disease in many crops (Bi et al. 2023; Amselem et al. 2011). The disease is widespread under favorable conditions, in particular cool temperatures and high humidity. In warmer conditions, *B. cinerea* switches to a survival mode and does not infect plants. Under severe stress conditions, growth is arrested (Basta et al. 2020; Zhang et al. 2023) and the fungus produces sclerotia, which are essential for long‐term survival (Williamson et al. 2007). Recently, it was discovered that passing through moderately high temperatures before being exposed to severely high temperatures primes the fungus to better cope with potentially lethal heat stress (Zhang et al. 2023). This priming effect involves enhanced production and accumulation of over 350 proteins, whereas these proteins are found to aggregate at severely high temperatures. One of the most prominent groups of proteins in this set is serine‐type peptidases, which are more highly expressed and enriched in the supernatant under priming conditions. Among them, BcPrm1, a S28 family serine peptidase, was found to be essential for a full priming response.

The connection between this group of serine‐type peptidases and heat adaptation, and specifically with priming, has so far only been described in *B. cinerea*. However, the role of other proteases in heat adaptation, particularly ATP‐dependent proteases, is well established in both prokaryotic and eukaryotic organisms (Meyer and Baker 2011; Maziak et al. 2021; Mahmoud and Chien 2018). For example, in *Pseudomonas aeruginosa* , a hierarchical function of a cascade of proteases was revealed in which the FtsH and ClpXP proteases play a primary role, and HslVU and Lon deploy a secondary response (Basta et al. 2020). *Lon* deletion mutants were found to be hypersensitive to severe, but not moderately high temperatures. In eukaryotes, the highly conserved ATP‐dependent mitochondria‐residing Lon protease is involved in proteostasis and maintenance of mitochondrial integrity and homeostasis (Rigas et al. 2009; Sundararajan Venkatesh et al. 2012; Li, Millar, et al. 2017; Pinti et al. 2016). As such, it is also associated with heat shock response (Adam et al. 2012; Puri and Karzai 2017), which is consistent with its roles as a chaperone that aids in proper protein folding and as a protease that degrades damaged proteins (Pinti et al. 2016; Gur and Sauer 2008; Li, Nelson, et al. 2017).

Another process involved in adaptation to stress is autophagy, the master of cellular bulk and selective recycling. Upon induction of autophagy, damaged proteins or organelles are sequestered into vacuoles or lysosomes, where they are further degraded by nonspecific proteases or other hydrolases (Nakatogawa et al. 2009; Mizushima and Komatsu 2011; Khan et al. 2012; Voigt and Pöggeler 2013a). Damaged organelles, such as mitochondria, can also be degraded by autophagy, then defined as mitophagy (Müller et al. 2015; Youle and Van Der Bliek 2012; Zhang et al. 2022). In addition, autophagy balances the synthetic and degradative modes of DNA polymerase γ, contributing to the stability and copy number dynamics of the mitochondrial genome (Medeiros et al. 2018). Deletion of autophagy regulatory genes in fungi has pleiotropic effects that vary according to species, and may include deformed mycelia (Voigt and Pöggeler 2013b; Ren, Liu, et al. 2018; Duan et al. 2013) and growth retardation (Khan et al. 2012; Duan et al. 2013; Kershaw and Talbot 2009; Liu, Xu, et al. 2016), reduced virulence, and defects in sporulation and appressoria maturation (Kikuma and Kitamoto 2011; Liu, Ning, et al. 2016).

We investigate the connections between the *B. cinerea* mitochondria‐residing BcLon1 protease, mitochondrial damage, and autophagy under optimal temperatures and heat stress. We show that under heat stress, but not under optimal conditions, deletion of *bclon1* induces mitochondrial malfunction and accelerates fungal cell death and the induction of autophagy. Blocking of autophagy in the background of *bclon1* deletion further intensified both phenomena. In addition, blocking of autophagy affected nuclei division and spore morphology, as well as mycelia growth and spore germination. Our results support a cytoprotective role for autophagy downstream of mitochondria‐driven death signals, possibly as a mechanism that promotes growth arrest and helps remove damaged cellular components.

## 2 Results

The mitochondria Lon1 protease is essential for protein quality control and maintenance of mitochondrial homeostasis. We hypothesized that conditions that disturb mitochondrial homeostasis, such as heat stress, activate downstream stress adaptation mechanisms, including autophagy, which helps cells to cope with the stress by removing damaged proteins and organelles. To test this hypothesis, we generated *bclon1* and *bcatg1* single and double mutants (Table S1) and tagged them with GFP‐ATG8 to detect the onset of autophagy and with mt‐mCherry to monitor mitochondrial structure.

### 2.1 Blocking of Autophagy by Deletion of *bcatg1*

**FIGURE 1:**
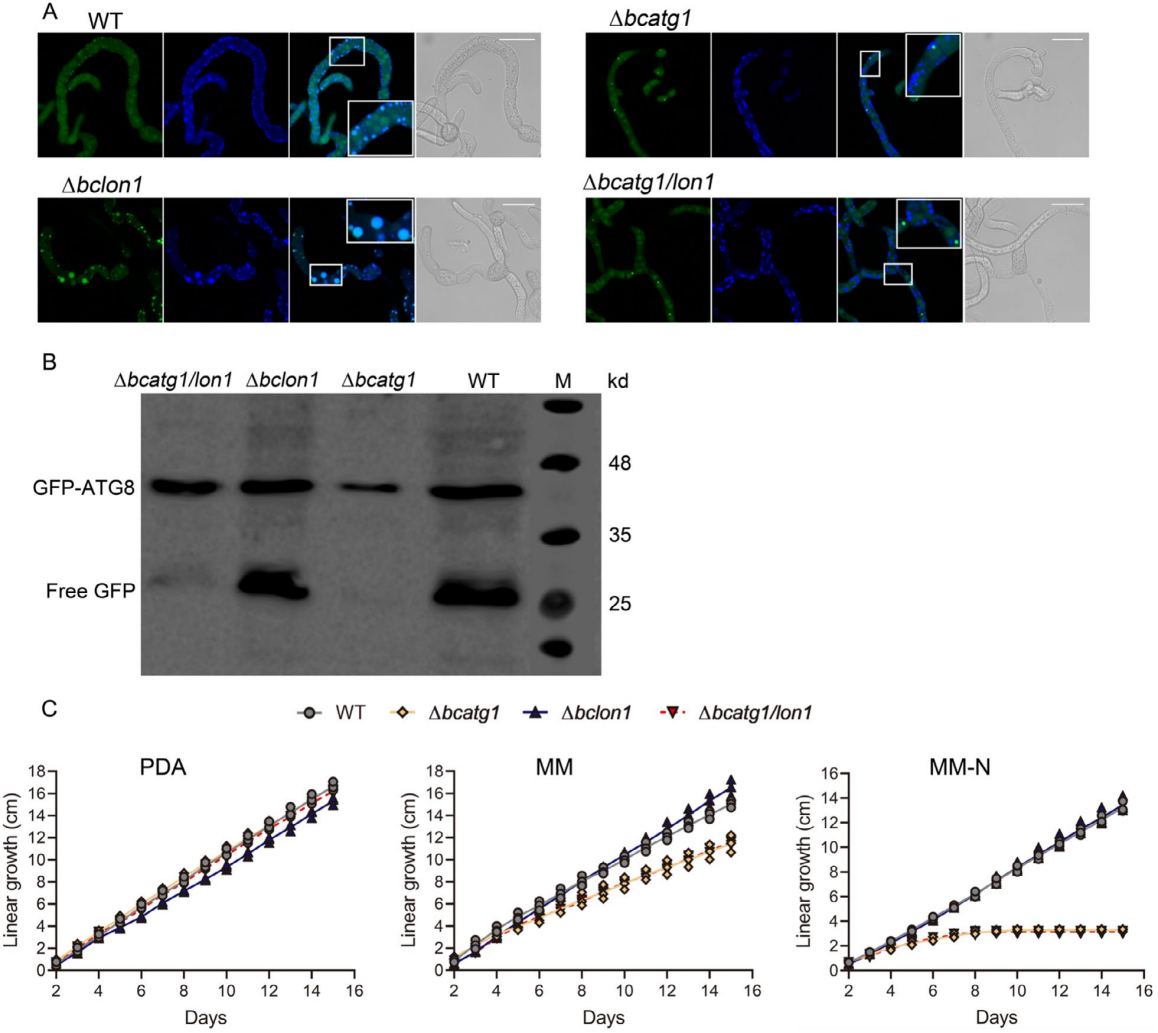
Autophagy is blocked by the deletion of *bcatg1*. (A) Monitoring of autophagy. Spores were incubated at 22°C for 6 h to produce germ tubes and then transferred to 29°C. PMSF and CMAC were used to inhibit vacuole hydrolases and stain vacuoles, respectively. Fluorescence microscopic images (left to right: GFP, DAPI, Merge, and DIC channels) of germ tubes were captured after incubation for 18 h at 29°C. Insets show accumulation of GFP in vacuoles (wild type and Δ*bclon1*), and blocking autophagy (Δ*bcatg1* and Δ*bcatg1/lon1*). Scale bars = 20 μm. (B) Western blot analysis. Spores were germinated in liquid G5 medium at 22°C for 6 h, and then samples were harvested, and proteins were extracted and analyzed by western blot with anti‐GFP antibodies. A 27 kD band of free GFP is expected when autophagy takes place. (C) Race tube assay experiments under rich nutrients (PDA), moderate starvation (MM), and severe starvation (MM‐N) conditions. The race tubes were incubated at 22°C, and linear growth of mycelium was recorded daily. Graphs represent three biological replications with overlaid individual data points.

To determine if autophagy is effectively blocked in the strains lacking *bcatg1*, we monitored the accumulation of GFP in vacuoles in strains expressing the GFP‐ATG8 fusion proteins, which is indicative of autophagy (Ren, Liu, et al. 2018; Nair et al. 2011; Torggler et al. 2017). For better visualization of the signal, we add the protease inhibitor PMSF, which blocks vacuole proteases and hence allows accumulation and detection of ATG8‐GFP in the vacuole. Spores were germinated on coverslips at 22°C, transferred to 29°C for 18 h, and then stained with CMAC (7‐amino‐4‐chloromethylcoumarin), which accumulates in the vacuole. In the wild type and Δ*bclon1* strains, the GFP and CMAC signals overlapped, indicating induction of autophagy (Figure 1A). In the Δ*bcatg1* strain and Δ*bcatg1/lon1* strains, there was no GFP signal in the vacuoles and a small number of intense green spots were observed in the cytoplasm, representing GFP‐AGT8 aggregates that attach to the vacuole but fail to fuse with the outer membrane due to the blocking of autophagy (Torggler et al. 2017). Likewise, clear autophagy was induced in the Δ*bclon1* strain by starvation condition (Figure S1). As expected, free GFP, another marker for autophagy induction (Nair et al. 2011; Torggler et al. 2017), was detected in protein extracts from the wild type and ∆*bclon1*, but not in the ∆*bcatg1* or ∆*bcatg1/lon1* strains (Figure 1B). To validate the cellular markers, we measured the growth rate of the strains under rich (PDA), moderately poor (MM) and poor (MM‐N) nutritional conditions (Figure 1C). All strains had a similar linear growth rate under PDA, while under MM, the growth rate of the ∆*bcatg1* and ∆*bcatg1/lon1* strains was reduced compared with the wild type. Under MM‐N, growth of the ∆*bcatg1* and ∆*bcatg1/lon1* strains stopped completely after 7 days. Collectively, these results confirmed that autophagy was blocked in the ∆*bcatg1* and ∆*bcatg1/lon1* strains.

### 2.2 Autophagy and the Lon1 Protease Both Contribute to *B. cinerea* Heat Adaptation

The optimal growth temperature for *B. cinerea* is around 22°C, with temperatures above 26°C generating heat stress and activating adaptation responses (Zhang et al. 2023). To test whether autophagy and BcLon1 are necessary for tolerance to heat stress, we compared the development of the different *B. cinerea* strains under optimal and moderately high temperatures.

**FIGURE 2:**
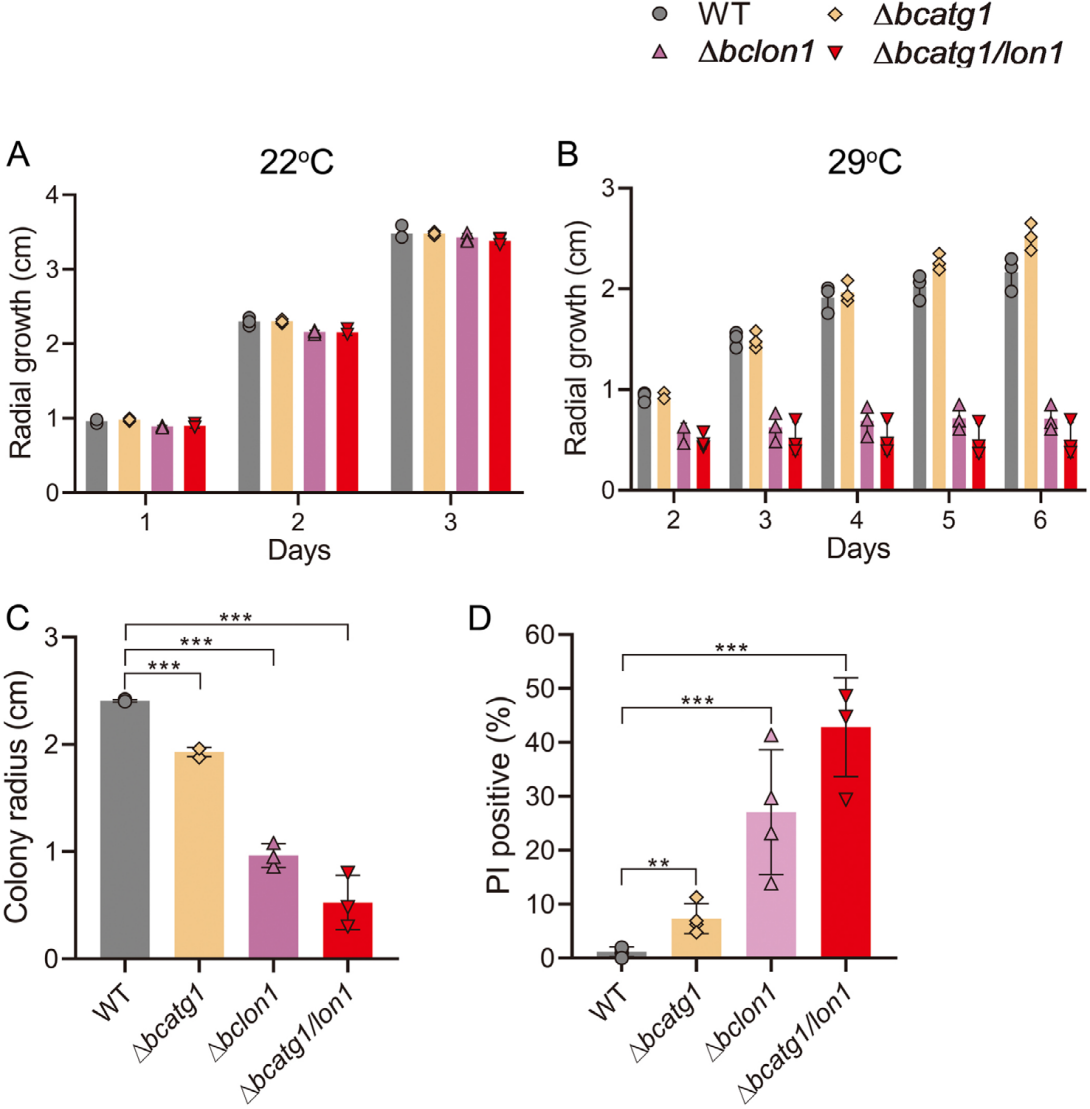
Autophagy and the Lon1 protease both contribute to *Botrytis cinerea* heat adaptation. (A, B) Cultures were initiated on PDA plates from mycelial plugs and the plates were incubated at 22°C and 29°C, and colony diameter was recorded daily. (C) Cultures were initiated on PDA from mycelial plugs. The plates were incubated at 32°C for 3 days and then to 22°C. After 3 days the cultures were stained with cotton blue to visualize the mycelia and colony diameter was recorded. (D) Spores were germinated at 22°C for 6 h, the germ tubes were transferred to 32°C for 24 h, stained with PI and the percent of PI‐positive (dead) germ tubes was determined. Graphs represent three (A–C) and four (D) biological replications with overlaid individual data points. Values are presented as the mean of replicates ± SD. Statistical differences were determined using unpaired two‐tailed Student's *t*‐test (**p < 0.01; ***p < 0.005).

Similar mycelia growth rate was detected in the four strains under optimal temperature (Figure 2A). At 29°C, the ∆*bclon1* and ∆*bcatg1/lon1* strains stopped growing on the second day of incubation, with a stronger effect in ∆*bcatg1/lon1* compared with ∆*bclon1* (Figure 2B). To test how blocking autophagy and deleting *bclon1* affect the recovery of mycelium from heat stress, mycelia plugs were placed on a PDA medium, incubated for 3 days at 32°C, and then transferred to 22°C for 3 days. Colony diameter was recorded as a measure of radial growth. Recovery of ∆*bcatg1* after heat stress was only slightly reduced compared with the wild type strain, whereas the ∆*bclon1* strain produced a much smaller colony, a phenomenon that was intensified in the *∆bcatg1/lon1* double mutant (Figure 2C and Figure S2A). Next, to test whether high temperature inhibits or kills the hyphae, we germinated spores at 22°C for 6 h, transferred them to 32°C for 24 h, and stained the resulting hyphae with PI. In line with the observed effect on colony recovery, wild‐type hyphae remained viable and only low levels of cell death were detected in the ∆*bcatg1*, while in the ∆*bclon1* and ∆*bcatg1/lon1* strains, close to 25% and 40% of the hyphae, respectively, were PI‐positive (Figure 2D and Figure S2B). Together, these results suggest that deletion of *bclon1* increases the sensitivity of hyphae to high temperatures and this phenomenon is intensified when combined with blocking of autophagy.

### 2.3 Deletion of *bclon1* Generates Mitochondrial Damage Under Heat Stress

**FIGURE 3:**
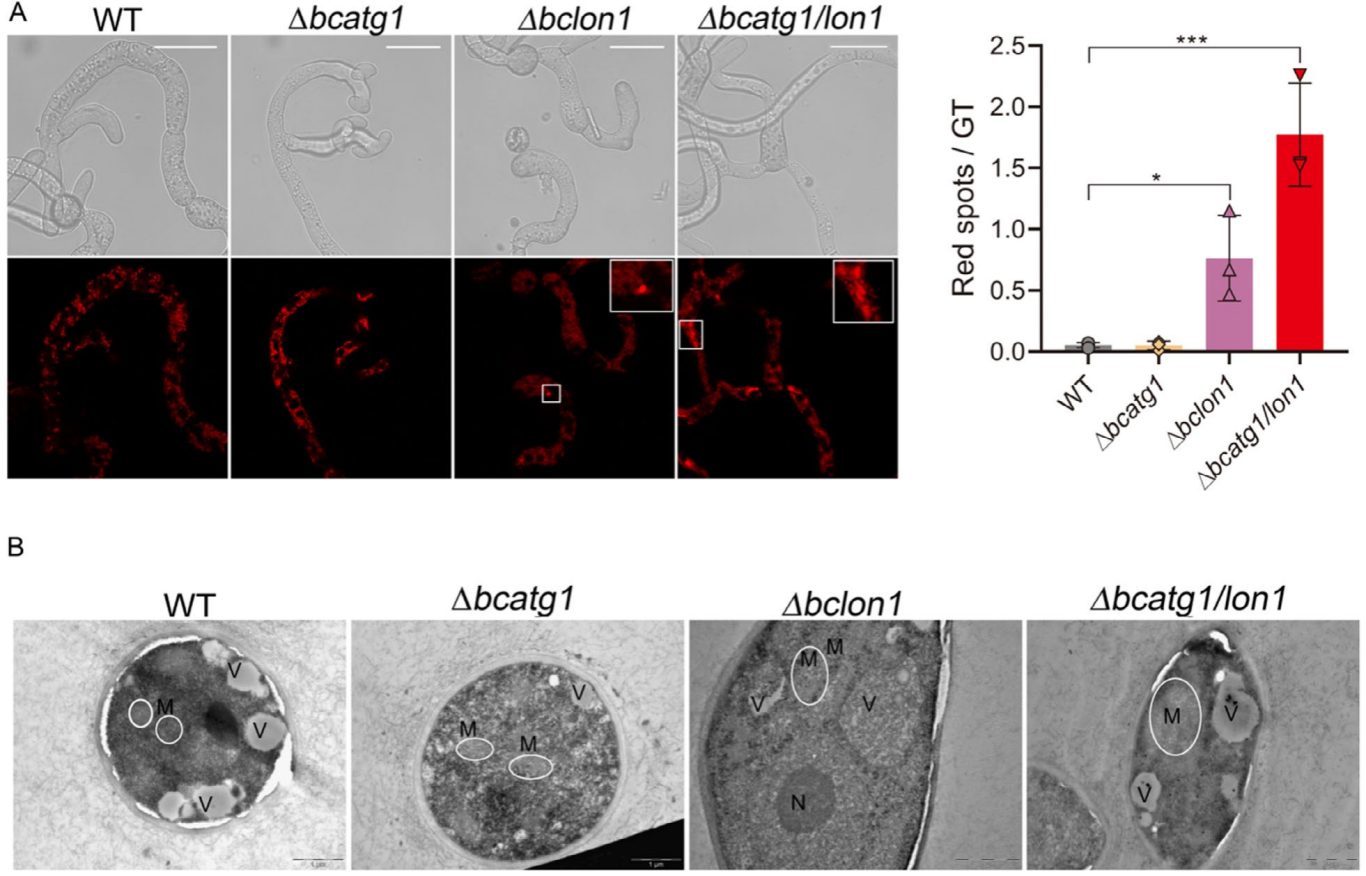
Deletion of *bclon1* causes more intense mitochondrial damage under heat stress. (A) Mitochondria morphology. Spores were germinated at 22°C for 6 h and the germ tubes were transferred to 29°C. Samples were removed after 18 h and images were captured with a fluorescent microscope using a rhodamine filter. Insets show accumulation of red spots (in ∆*bclon*1 and ∆*bcatg*1*/lon*1), which were turned out to be mCherry aggregates outside the mitochondria (Figure S4). Scale bars = 20 μm. The graph shows average number of red spots per germ tube from three biological replications with overlaid individual data points. Values are presented as the mean of replicates ± SD. Statistical differences were determined using unpaired two‐tailed Student's *t*‐test (*p < 0.05; ***p < 0.005). (B) Germ tubes were collected after incubation for 18 h at 29°C, processed, and scanned by transmission electron microscopy (TEM). White line circles mark mitochondrial boundaries. M, mitochondria; N, nucleus; V, vacuole. Scale bars = 1 μm.

Lon1 is necessary to maintain mitochondrial homeostasis (Sundararajan Venkatesh et al. 2012; Pinti et al. 2016, 2015; Li, Nelson, et al. 2017). To test the effect of high temperature on the mitochondria in the different strains, spores were germinated at 22°C, transferred to 29°C for 18 h, and samples were inspected with a confocal microscope. The mitochondria of all four strains were punctate when the fungi were cultured at 22°C (Figure S3A). However, at 29°C we detected the appearance of enlarged red spots in hyphae of the ∆*bclon1* and ∆*bcatg1/lon1* strains (Figure 3A), which were not present when cultured at 22°C. To better define the nature of these bodies, we stained the hyphae with mitoTracker green, which stains mitochondria. These analyses showed that the red spots did not co‐localize with the green mitoTracker signal (Figure S4). We interpreted this result as indicative of mCherry protein that was not delivered into the mitochondria and formed aggregates outside the mitochondria due to malfunction of the mitochondria delivery machinery (Waegemann et al. 2015; Demishtein‐Zohary and Azem 2017). Accordingly, we used the number of enlarged red spots to estimate mitochondrial damage. The average number of enlarged red spots in hyphae of the ∆*bcatg1/lon1* was more than twofold higher than in ∆*bclon1*, while no red spots were evident in hyphae of the wild type and ∆*bcatg1* strains (Figure 3A). Hence, deletion of *bclon1* sensitizes mitochondria to heat stress, and this phenomenon is intensified when combined with blocking of autophagy.

To further quantify mitochondrial damage in the ∆*bclon1* strain, we measured the levels of mitochondrial membrane potential (MMP) and reactive oxygen species (ROS). We germinated spores for 10 h at 22°C (control) and 29°C (heat stress), and then stained them with Mito‐ID and DHR123, used to measure MMP and ROS, respectively. For MMP, strong red signals were detected in the wild type, while these signals faded in the ∆*bclon1* strain, both at 22°C and 29°C (Figure S5A). In addition, the wild‐type strain showed higher ratios of red/green signal than the ∆*bclon1* strain, indicating healthier mitochondria (Figure S5B). For ROS, similar levels were detected in the wild type and ∆*bclon1* strains when fungi were cultured at 22°C, while at 29°C the ROS levels in the ∆*bclon1* strain were significantly higher than in the wild‐type strain (Figure S5C,D).

To further characterize the effect of autophagy and deletion of *bclon1* on the mitochondria, we examined hyphae with a transmission electron microscope (TEM). When the fungi were cultured at 22°C, in all four strains the mitochondria outer membranes were well defined and the cristae were visible (Figure S3B), which is indicative of healthy mitochondria. At 29°C, the mitochondria of the ∆*bclon1* and ∆*bcatg1/lon1* strains were hardly visible, and their outer membrane and cristae were fuzzy (Figure 3B). Additionally, the *∆bcatg1/lon1* strain had swollen mitochondria. These TEM results support the confocal microscopy analyses, and collectively these results show that deletion of *bclon1* increases mitochondrial damage under heat stress. The damage to mitochondria caused by deletion of the *bclon1* was intensified by blocking autophagy, suggesting that autophagy partially alleviates heat stress damage downstream of *bclon1*.

### 2.4 Deletion of *bclon1* Results in Earlier and More Intense Autophagy Under Heat Stress

**FIGURE 4:**
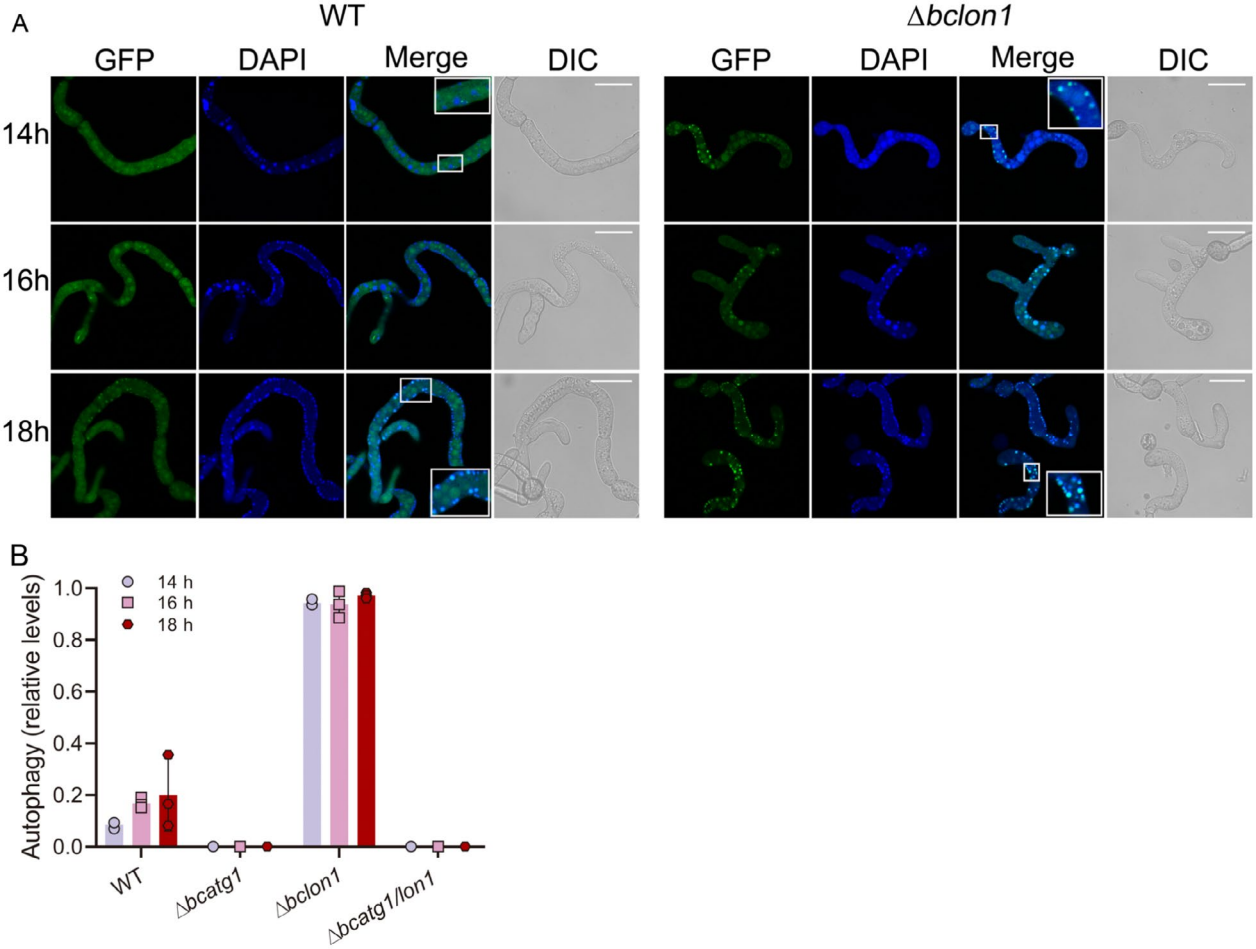
Deletion of *bclon1* causes earlier and more intense autophagy under heat stress. (A) Spores were germinated at 22°C for 6 h and the germ tubes were transferred to 29°C. Samples were collected after 14, 16, and 18 h, the vacuoles were stained with CMAC and images were captured using a fluorescent microscope with GFP and DAPI. Insets show areas of hyphae with vacuoles before (CMAC only, wt 14 h) or after (CMAC and GFP) induction of autophagy. Left—wild type, right—∆*bclon1*. Scale bars = 20 μm. (B) Relative levels of autophagy. The number of vacuoles with CMAC signal and vacuoles with overlapped GFP and DAPI signals were counted. The ratio between autophagy vacuoles with overlap signals (autophagy) and vacuoles with only CMAC signal (no autophagy) was calculated. Graphs represent three biological replications with overlaid individual data points.

To check the onset and level of autophagy under heat stress, we germinated spores of GFP‐ATG8 expressing strains at 22°C for 6 h and then transferred them to 29°C. Samples were removed after 14, 16, and 18 h of incubation at 29°C and treated with PMSF (a proteases inhibitor) to inhibit vacuole hydrolases. The vacuoles were stained with CMAC, and the samples were examined with a confocal microscope. When the fungi were incubated at 22°C (control), in all four strains the GFP signal remained mainly in the cytoplasm throughout the incubation period (Figure S6). When fungi were incubated at 29°C, the GFP signal in the wild‐type strain was localized only in the cytosol until 16 h, and at 18 h it was detected in 20% of vacuoles, whereas in the ∆bclon1 strain 90% of the vacuoles were GFP‐positive already after 14 h of incubation at 29°C (Figure 4A,B). In the absence of autophagy (∆bcatg1 and ∆bcatg1/lon1), the GFP signal could not be detected in vacuoles under any conditions (Figure 1A). These results confirmed induction of earlier and more intense autophagy in the ∆*bclon1* strain under heat stress.

### 2.5 Blocking of Autophagy Affects Nuclei Number and Cell Cycle

Spores of all four strains reached around an 80% germination rate after 3 h at 22°C (Figure S7A). At 32°C, the germination rates of all strains were reduced compared with 22°C, though the effect on the different strains was not uniform; the ∆*bclon1* strain was the most severely inhibited, attaining less than 10% germination after 8 h, whereas the germination rate of the ∆*bcatg1* was close to 60%—much higher than the wild type and the Δ*bcatg1/lon1* double mutant, both of which were close to 35% (Figure S7B). The observed reduced germination and growth rate of the ∆*bclon1* strain under high temperatures suggested that the ability of this strain to cope with heat stress is compromised. To further check whether high temperature results in higher death rates of ∆*bclon1* spores, we kept dry spores at 22°C and 29°C and compared the levels of cell death following staining with propidium iodide (PI). When incubated at 22°C, spore death rates of all strains were close to zero during the first 21 days. However, after 28 days there was a sharp increase in the death rates of ∆*bcatg1* and ∆*bcatg1/lon1* spores to around 20%, whereas those of the wild type and the ∆*bclon1* strains remained close to zero (Figure S7C). Similar results were obtained at 29°C, albeit with earlier (14 days) and higher death rates (Figure S7D). Hence, the deletion of *bclon1* does not lead to enhanced cell death in spores at the above temperatures. On the other hand, blocking of autophagy resulted in higher germination rates and increased levels of spore death under high temperatures. These results suggest that blocking of autophagy impairs the regulation of growth arrest at high temperatures.

**FIGURE 5:**
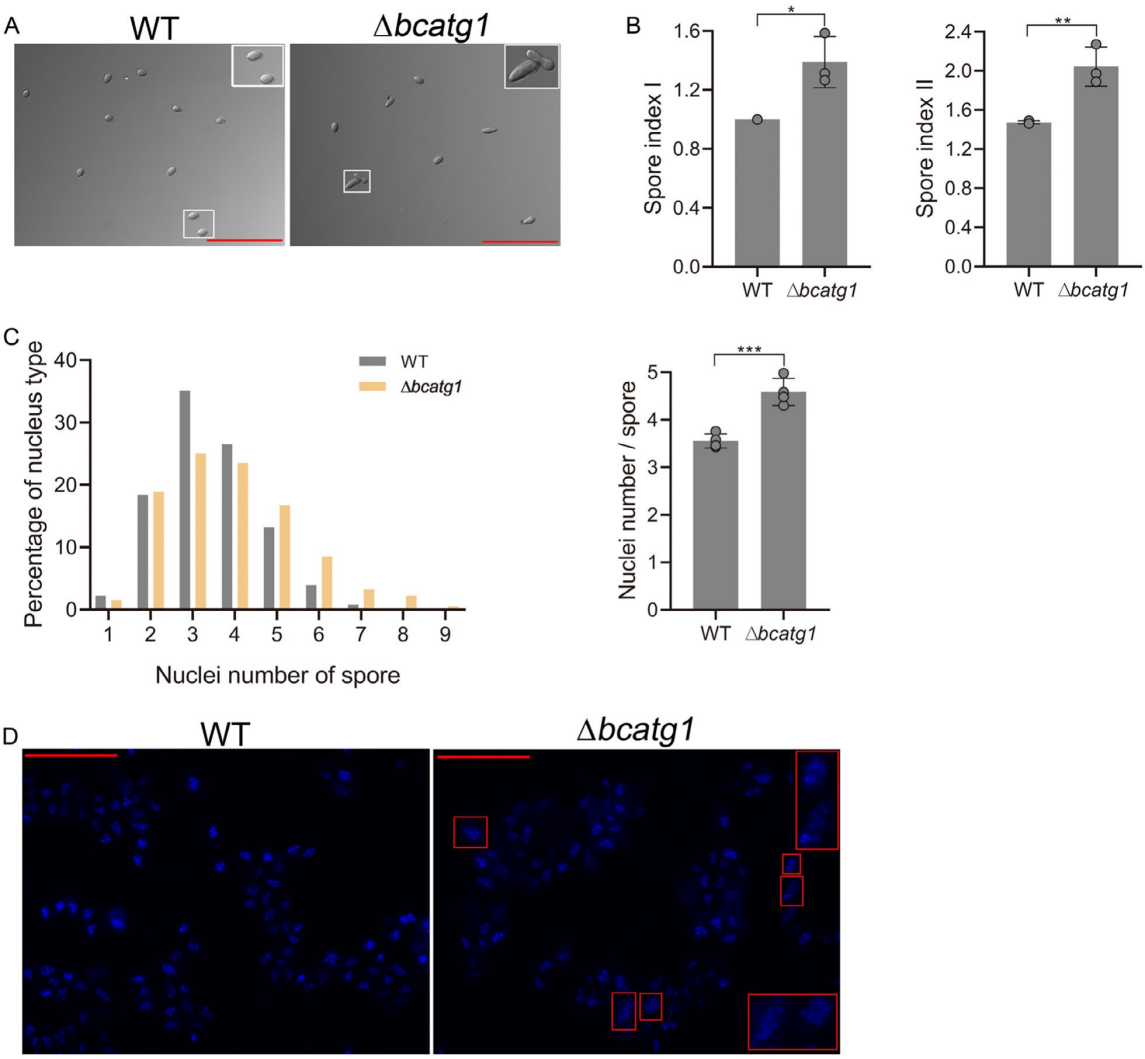
Blocking of autophagy affects nuclei number and cell cycle. (A, B) Spore morphology and spore indexes. Fungi were cultured on PDA for 7 days, spores were collected, images were captured (A) and spore index I (spore area relative to wild type) and II (ratio of spore length and width) were determined. Insets show spore morphology in the wild type and ∆*bcatg1* strains. (C, D) Quantification of nuclei number. Spores were fixed in ethanol, stained with Hoechst, and images were captured using a fluorescent microscope with a DAPI filter. The number of nuclei per spore was counted and the average number of nuclei per spore was calculated using SCAN. Red lines mark the enlarged spores with a high number of nuclei. Graphs represent three (B) and four (C) biological replications with overlaid individual data points. Values are presented as the mean of replicates ± SD. Statistical differences were determined using unpaired two‐tailed Student's *t*‐test (*p < 0.05; **p < 0.01; ***p < 0.005). Scale bars = 100 μm.

As a common stress adaptation strategy, growth arrest was reported in different organisms under stress conditions (Puente et al. 2014; Mokas et al. 2009; Hwang et al. 2018). In the budding yeast *Saccharomyces cerevisiae* , inhibition of autophagy results in exaggerated filamentous growth, while overexpression of autophagy‐related genes inhibits this growth (Ma et al. 2007). Further study showed that wild‐type yeasts preferentially arrest in G_1_/G_0_ in response to starvation, while yeasts with autophagy blocking show a significantly higher percentage of cells in G_2_/M (An et al. 2014). In accordance with these reports and our results, we hypothesized that the higher rates of spore death in the null autophagy strain under heat stress are the consequence of cell cycle impairment. This possibility is supported by the elongated spores of the ∆*bcatg1* strain (Figure 5A), which is in line with previous reports (Ren et al. 2017).

To further define this phenotype, we compared the dimensions of wild type and ∆*bcatg1* spores using two indexes: (1) Index I was defined as the area ratio between mutant and wild type spores; (2) Index II was defined as the ratio between spore length and width. Both indexes proved to be larger in the ∆*bcatg1* strain than in the wild‐type strain (Figure 5B). To check whether the changes in the ∆*bcatg1* spore dimensions reflect defects in cell cycle regulation, we counted and compared the number of nuclei in wild type and ∆*bcatg1* mutant spores using two methods: (1) manually counting the number of nuclei in each spore to generate distribution graphs; and (2) automatically counting the total numbers of nuclei and spores using the SCAN software (Shlezinger et al. 2014) and determining the average number of nuclei per spore. Both parameters showed an increased number of nuclei in spores from the ∆*bcatg1* strain, with the wild‐type spores having an average of 3.5 nuclei per spore and a distribution between 1 and 7 nuclei compared with 4.5 nuclei per spore in the ∆*bcatg1* deletion strain and a distribution between 1 and 9 nuclei (Figure 5C,D).

The enlarged spores and increased nuclei number observed in the ∆*bcatg1* strain indicated that deletion of *bcatg1* affected cell cycle regulation. To determine whether this effect was associated with blocking autophagy, or whether it was a specific effect of the deletion of *bcatg1*, we generated deletion mutants of two additional autophagy‐related genes, *bcatg4* and *bcatg9*. We used the GFP‐ATG8 marker to verify that autophagy was blocked in both strains (Figure S8A) and then determined spore indexes. Both strains had elongated spores (Figure S8B), and their spore indexes were higher than those of the wild‐type spores (Figure S8C). Similar to ∆*bcatg1*, spore germination of ∆*bcatg4* and ∆*bcatg9* was also enhanced at 32°C compared with the wild type (Figure S8D), confirming that blocking autophagy relieves the heat‐mediated inhibition of spore germination. This observation is in accordance with the increased spore size in the *bcatg* deletion strains and further supports a role for autophagy in cell cycle regulation.

## 3 Discussion

Heat adaptation is multilayered, involving a coordinated response of general and heat‐specific cellular systems and processes. Here, we investigated the involvement of autophagy and mitochondria proteostasis in the adaptation of *B. cinerea* to mild heat stress. At optimal temperature, the disruption of autophagy caused growth retardation under starvation conditions and accelerated aging in spores (Figure S7C,D), while a Δ*bclon1* mutant had no visible phenotype, suggesting no interaction between the two processes in adaptation to nutrient stress. However, the Δ*bclon1* mutant exhibited higher mitochondrial damage (higher ROS level and compromised mitochondrial membrane potential) and reduced spore germination under heat stress. More interestingly, further deletion of *bcatg1* caused additional growth defects (Figure 2B–D) and mitochondrial malfunction (Figure 3) than the Δ*bclon1* single mutant. In addition, the Δ*bclon1* strain showed an earlier and more intense autophagy than the wild‐type strain (Figure 4). Collectively, these results suggest both Lon1 and autophagy contribute to the heat stress response in a coordinated manner.

Independent of temperature, blocking of autophagy resulted in growth arrest under nutrient limiting conditions, as has been well established (Khan et al. 2012; Ren, Liu, et al. 2018; Liu, Xu, et al. 2016; Ren, Sang, et al. 2018). Autophagy mutant strains also exhibited enlarged spores with an increased number of nuclei, suggesting mis‐regulation of the spore cell cycle (Gonzalez et al. 2018), which might explain the enhanced germination and mycelium growth of *bcatg* mutants under heat stress. Thus, we interpret the presence of additional nuclei and increased conidial size in the Δ*bcatg*1 mutant not as a result of conidia being born multinucleate, but as a consequence of defective regulation of conidial maturation. The mutant conidia appear to continue growing and undergoing nuclear division beyond the normal developmental timeline, delaying dormancy and cell cycle arrest. Consistent with this, blocking autophagy in *Fusarium oxysporum* by deleting the *foatg8* gene has been shown to result in an increased number of nuclei (Corral‐Ramos et al. 2015). Additionally, Atg8‐mediated nuclei degradation has been proposed in several studies (Liu and Yao 2012; Dou et al. 2016). Our results support a connection between autophagy and cell cycle regulation and growth, and show that in addition to autophagy's pivotal role in recycling of damaged cellular components, it might be necessary for proper execution of growth arrest in response to heat stress.

Lon1, a serine AAA+ (ATPases associated with various cellular activities) protease localized to the mitochondria, is part of the protein quality control machinery that maintains mitochondria proteostasis and homeostasis (Tatsuta and Langer 2008). Specifically, Lon1 controls selective turnover of non‐assembled or misfolded proteins that accumulate in the mitochondrial matrix and is necessary for proper mitochondrial function (Quirós et al. 2015). In fungi, deletion of *lon1* has been reported to destabilize the mitochondria, resulting in developmental defects such as reduced growth in yeasts (Suzuki et al. 1994; Erjavec et al. 2013) and heat‐dependent ascospore formation and lethality in the filamentous fungus *Podospora anserina* (Adam et al. 2012). The *B. cinerea* Δ*bclon1* deletion strain had a normal phenotype under optimal temperature, but at high temperatures growth was arrested and spore germination was severely retarded. Moreover, earlier and more intense autophagy was observed in the Δ*bclon1* mutant, which also exhibited intensified mitochondrial damage. Importantly, growth arrest, cell death level, and mitochondrial malfunction were more extreme in the Δ*bcatg1/lon1* double mutant, despite a lack of effect (life span was unaffected in Δ*bclon1*, mitochondrial damage was unaffected in Δ*bcatg1*) or even opposite effects (enhanced versus retarded growth and germination in the Δ*bcatg1* and Δ*bclon1*, respectively) in the single mutants. Collectively, these results indicate that BcLon1 and autophagy both contribute to heat adaptation and support the role of autophagy as a protective machinery, either downstream or in parallel of mitochondria‐derived stress signals. Our data suggest that autophagy has a dual function, in which it provides a cytoprotective function as well as necessary for regulation of the growth arrest under heat stress.

## 4 Experimental Procedures

### 4.1 Fungal Cultures

*Botrytis cinerea* B05.10 and derived transgenic strains were routinely cultured on potato dextrose agar (PDA; Acumedia) at 22°C under continuous fluorescent light supplemented with near UV light. Additional media used for specific experiments included potato dextrose broth (PDB; Acumedia), malt medium (5 g glucose, 15 g malt extract, 1 g peptone, 1 g casamino acids), SH medium (205.4 g sucrose, 0.6 g Tris–HCl, 0.13 g (NH_4_)_2_HPO_4_, 10 g agar, and bring to pH 6.5 with HCl), minimal medium (0.5 g KCl, 2 g NaNO_3_, 1 g K_2_HPO_4_, 0.5 g MgSO_4_(H_2_O)_7_, 0.01 g FeSO_4_(H_2_O)_7_, 30 g glucose, 12 g agar, and bring to pH 6.9 with HCl), MM‐N (minimal medium without NaNO_3_), LB medium (10 g Bacto tryptone [Difco], 5 g yeast extract [Difco], 5 g NaCl), and GB5‐Glc medium (Gamborg B5 with vitamins and 2% glucose; Duchefa Biochemie).

### 4.2 Generation of *B. cinerea* Mutant Strains

To monitor mitochondria morphology and autophagy, we constructed a cassette with a GFP‐tagged *bcatg8* (BCIN_02g02570) driven by the *Aspergillus nidulans OliC* promoter, an mCherry fused to the mitochondrial signal of the BCIN_02g02750 gene driven by the *B. cinerea* H2B promoter, and a hygromycin resistance cassette. The wild type and a nourseothricin resistance *bclon1* deletion strain (∆*bclon1*) were transformed with the plasmid POA‐∆BCgpd‐GFP‐ATG8‐Mt_mCherry containing the GFP‐*bcatg8*/mt‐mCherry cassette, which was inserted at the 3′ end of the *bcgpd* gene to ensure uniformity of the transgenic strains, stable expression, and prevention of possible side effects. To generate *bcatg1* deletion (∆*bcatg1*) and *bcatg1/lon1* double deletion (∆*bcatg1/lon1*) strains, the wild type (for generation of ∆*bcatg1*) and ∆*bclon1* (for generation of ∆*bcatg1/lon1*) were transformed with the plasmid PTZ‐∆ATG1‐GFP‐ATG8‐Mt_mCherry containing the GFP‐*bcatg8*/mt‐mCherry cassette flanked by 500 bp of *bcagt1* 5′ and 3′ genomic regions. *bcatg4* and *bcatg9* deletion strains (Δ*bcatg4* and Δ*bcatg9*) were generated by replacing the entire open reading frame (ORF) of each gene with the plasmid PTZ‐∆ATG4‐GFP‐ATG8 or PTZ‐∆ATG9‐GFP‐ATG8 containing a GFP‐*bcatg8* cassette flanked by 500‐700 bp of *bcagt4* or *bcatg9* 5′ and 3′ genomic regions and a hygromycin resistance cassette. For PI, Mito‐ID and DHR123 staining‐related experiments, *bcatg1* and *bcatg1/bclon1* deletion strains (Δ*bcatg1#* and Δ*bcatg1/bclon1#*) were generated by replacing the *bcatg1* gene with the plasmid PTZ‐∆ATG1‐Hyg containing a hygromycin resistance cassette flanked by 500 bp of *bcagt1* 5′ and 3′ genomic regions. Details of all strains and plasmids are provided in Tables S1 and S2.

Genetic transformation of *B. cinerea* was performed as previously described (Ma et al. 2017). At least 10 isolates were obtained for each strain, and DNA was extracted from each colony and analyzed by PCR to verify integration of the construct at the desired genetic locus. Homokaryotic strains were obtained by single spore isolation using a Sporeplay dissection microscope (Singer Instruments, UK). Derived colonies were analyzed by PCR to verify that the strain is homokaryotic at the desired locus, and additional rounds of single spore isolation were performed in cases of impurity. Details of all primers are provided in Table S3. At least four separate single spore isolates were obtained for each strain.

### 4.3 Protein Extraction and Western Blot Analysis

Fungi were grown as described in 90‐mm Petri dishes containing 20‐mL liquid medium with agitation at 100 rpm. The mycelium was harvested, transferred into liquid nitrogen, and then lyophilized overnight. Dried mycelium was ground to a fine powder using a tissue grinder for 1 min at 30 Hz (Tissuelyser; QIAGEN) and aliquots of the mycelium powder were suspended in 300–400‐μL lysis buffer (0.2 M NaOH, 5% β‐mercapto‐ethanol, ddH_2_O) and incubated on ice for 30 min. Meanwhile, an equal volume of lysis buffer was titrated with HCl 16% to pH 9, and after incubation, the pH of the samples was neutralized by titrating with an equal volume of HCl. The lysate was centrifuged for 6 min at 6000 *g*, the supernatant was transferred to a fresh tube, and protein concentration was determined using a Bradford reagent. Before gel electrophoresis, loading buffer was added to equal amounts of proteins, and the samples were incubated at 95°C for 3 min. Proteins were separated by running on 10% SDS (sodium dodecyl sulfate)‐PAGE and blotted at 100 V onto a 0.45‐μm nitrocellulose membrane (Bio‐Rad) for 1 h at room temperature. The membranes were incubated for 30 min in 5% skim milk in TBST (Tris‐buffered saline with Tween 20: 50 mM Tris–HCL [pH 7.5], 200 mM NaCl, 0.05% [vol/vol] Tween 20) at room temperature with shaking, then hybridized for 1 h with the primary antibody (1:1000 dilution), and then for 30 min with a horseradish peroxidase‐conjugated secondary antibody (1:5000 dilution). The resulting immune complexes were visualized with Enhanced Chemiluminescence (ECL) (Pierce, Rockford): an equal volume (9 mL for two membranes) of Solution A (100 mM Tris, pH 8.5, 5.4 mM H_2_O_2_, ddH_2_O) and Solution B (100 mM Tris pH 8.5, 2.5 mM Luminol, 0.4 M P coumaric, ddH_2_O) were mixed in a light‐protected tube, the solution was added to the membrane, incubated 1–3 min at room temperature with shaking, and the membrane was incubated with a film for variable lengths of time before developing.

### 4.4 Microscopy

Fluorescent and light microscopy were performed with a Zeiss Axiovision imager M1 microscope. Differential interference microscopy (DIC) was used for bright‐field images. Confocal microscopy was performed with Zeiss Laser Scanning Microscopy 780 and transmission electron microscopy (TEM) with a JEM‐1400Plus TEM (JEOL, Japan).

### 4.5 Growth Assays

#### 4.5.1 Radial Growth

A 4‐mm mycelium plug was cut from the edge of a 2‐day‐old colony and placed on PDA in the center of a 50‐mm Petri dish. The plates were incubated at the indicated temperature, and colony diameter was measured daily, starting from the first or second day after inoculation. Each experiment was performed with three replications and was repeated at least three times.

#### 4.5.2 Race Tubes

Race tubes were used to measure linear growth rate over extended (longer than 5 days) periods of time. A sterile disposable 25‐mL plastic pipette was filled with sterile molten medium and half of the amount was slowly released, leaving 13 mL of medium inside the pipette. The tip was closed, and the pipette was gently laid horizontally starting with the tip to avoid wetting the cotton plug at the other end of the pipette. After the medium was solidified, a hole located close to the cotton‐plugged end was cut with a hot blade, 5 μL of spore suspension (10^6^ spores/mL) was placed at the edge of the medium and the hole was sealed with a strip of Parafilm. The tubes were incubated horizontally at 22°C and growth was recorded daily. Each experiment was performed with three replications and repeated at least three times.

### 4.6 Spore Morphology

Spores were collected by washing plates with sterile distilled water, and spore density was adjusted to 5 × 10^5^ spores/mL. A droplet of 20 μL of spore suspension was placed on a coverslip, and images were captured using a light microscope. Spore length and width were measured using the Axiovision microscope software, and the area of spores was measured with the Image J software. The average area of wild‐type spores was used to calculate spore index I, defined as the area of spore of a specific strain relative to the average area of wild‐type spores. Spore index II was calculated as the ratio between spore length and width. Each experiment was repeated at least three times with more than 100 randomly selected spores per treatment in each experiment.

### 4.7 Number of Nuclei in Spores

Spores were collected into 70% ethanol, and the density was adjusted to 5 × 10^5^ spores/mL. A droplet of 20 μL of spore suspension was placed on a coverslip, the ethanol was allowed to evaporate, the nuclei were stained by incubating the spores for 15 min in 50 μg/mL Hoechst 33342 (Thermo Fisher), and samples were examined with a fluorescent microscope using a DAPI filter. To determine the specific number of nuclei per spore, nuclei were manually counted in more than 600 spores per treatment. To determine the average number of nuclei per spore, the total number of nuclei and total number of spores were determined using the SCAN software (Shlezinger et al. 2014) by counting at least 600 spores per treatment. Each experiment was repeated four times.

### 4.8 Spore Germination and Cell Death

#### 4.8.1 Spore Germination

Spores were collected into PDB and density was adjusted to 5 × 10^5^ spores/mL. A 20‐μL droplet of spore suspension was placed on a microscope coverslip, incubated under the desired conditions, and samples were then examined using a light microscope. Each experiment was repeated at least four times, each time with over 300 spores that were randomly selected per treatment.

#### 4.8.2 Spore Cell Death

Spores were collected following incubation under desired conditions and stained for 15 min with 10 μg/mL solution of propidium iodide (PI; Sigma‐Aldrich, USA). Samples were examined using a fluorescent microscope with a rhodamine filter, and the number and ratio of dead spores (PI‐positive) were determined. Each experiment was repeated at least three times, with over 300 spores that were randomly selected per treatment.

### 4.9 Fungal Recovery After Heat Stress

#### 4.9.1 Recovery Assays

A 4‐mm mycelium plug was cut from the edge of a 2‐day‐old colony and placed on PDA in the center of a 50‐mm Petri plate. The plates were incubated in a light‐protected incubator at 32°C for 3 days and then transferred back to 22°C for recovery from the heat stress. After 3 days of incubation at 22°C, the colonies were stained with cotton blue for visualization of the mycelium, washed extensively with water, and colony diameter was measured. Each experiment was performed with three replications and repeated at least four times.

#### 4.9.2 Cell Death Assays

Spores were collected into PDB, and the density was adjusted to 5 × 10^4^ spore/mL. A 20‐μL droplet of spore suspension was placed on a microscope coverslip; the samples were incubated for 6 h at 22°C to allow spores to germinate and then incubated at 32°C for 24 h. The PDB medium was removed from the coverslip, and the mycelium was stained by suspending the samples in 10 μg/mL PI for 30 min. Samples were examined with a fluorescent microscope using the rhodamine filter, and the proportion of PI‐positive hyphae was calculated. Each experiment was performed with three replications per treatment and repeated at least three times.

### 4.10 Measurement of MMP and ROS Level

For ROS, spores were collected following incubation under desired conditions and stained for 15 min with 5 μg/mL solution of DHR123 (Thermo Fisher, USA). For MMP, samples were stained in a light‐protected tube with 1:50 solution of Mito‐ID (Enzo, USA). Samples were examined with a fluorescent microscope using the appropriate filters. Fluorescence signal intensity was quantified using ImageJ. Each experiment was repeated at least three times, with over 200 germ tubes that were randomly selected per treatment.

### 4.11 Monitoring of Autophagy and Mitochondria Morphology

#### 4.11.1 Confocal Microscopy

Spores from 7‐day‐old cultures were collected into G5 liquid medium, and the density was adjusted to 5 × 10^4^ spores/mL. A 20 μL droplet of spore suspension was placed on a microscope coverslip; the samples were incubated for 6 h at 22°C to allow spores to germinate and then transferred to 29°C for the desired amount of time. One hour before the end of incubation at 29°C, 1 μL of 10 μM CMAC was added to an Eppendorf tube containing 94 μL G5 liquid medium. The tube was incubated at 37°C for 30 min, and then 5 μL of 100 mM PMSF was added and mixed. The culture medium was gently removed from the coverslip, 20 μL of pre‐warmed PMSF/CMAC solution was added, and the samples were incubated for another 30 min at 29°C. Fresh G5 liquid medium with 5 μM PMSF was added at the end of the incubation, and the samples were analyzed by a confocal microscope using the GFP and DAPI filters. The experiment was repeated three times, with over 200 germ tubes that were randomly selected per treatment.

#### 4.11.2 TEM

Spores from 7‐day‐old cultures were collected into G5 liquid medium, and the density was adjusted to 10^6^ spores/mL. A 20 μL droplet of the spore suspension was placed on G5 solid medium in a Petri dish, incubated for 6 h at 22°C, transferred to 29°C for 18 h, and then 20 μL of 5 mM PMSF was placed on the top of young mycelium. Mycelial samples were collected into a 1.5 mL Eppendorf tube, fixed in 3.5% (v/v) glutaraldehyde in PBS, and then rinsed and postfixed in 1% (v/v) OsO4 in PBS. The samples were washed several times in PBS, stained with uranyl acetate, and then dehydrated by submerging in cold ethanol followed by acetone. The dry samples were embedded in Agar100 epoxy resin (Agar Scientific), cut to thin sections, and then treated with uranyl acetate/lead citrate. The samples were examined with a JEM‐1400Plus TEM (JEOL, Japan). Selected photographs represent images common to each treatment.

### 4.12 Data Analysis

Statistical analyses were performed with IBM SPSS Statistics package 22.0. The statistical significance between means of treatments was determined by Student's *t*‐test (two‐tailed *t*‐test). Graphs were generated using the Prism software (GraphPad 8.0). In all graphs, results represent the mean values of at least three independent experiments, each with at least three replications per treatment. Details of statistical analyses are presented in the figure legends.

## Author Contributions

**Mingzhe Zhang:** conceptualization, methodology, data curation, formal analysis, validation, investigation, visualization, writing – original draft, writing – review and editing. **Liang Ma:** methodology, conceptualization, investigation. **Zhiqun Lyu:** investigation. **Naomi Kagan Trushina:** investigation. **Amir Sharon:** conceptualization, methodology, data curation, supervision, formal analysis, funding acquisition, project administration, resources, writing – review and editing.

## Conflicts of Interest

The authors declare no conflicts of interest.

## Supporting information


**Figure S1.** Induction of autophagy by starvation. Spores were germinated at 22°C in PDB on coverslip for 6 h, then the PDB was replaced with fresh PDB (wt and Δ*bclon1* mutant) or with minimal medium (only Δ*bclon1* mutant) and the slides were incubated at 22°C for additional 18 h after which the hyphae were stained with CMAC. Images were captured using a fluorescent microscope with DAPI (for detection of CMAC staining) and GFP (for detection of ATG8‐GFP) filters. Scale bar = 10 μm.

**Figure S2.** Effect of autophagy and *bclon1* on cell death and survival of mycelia and germ tubes. (A) Cultures were initiated on PDA from mycelial plugs. The plates were incubated at 32°C for 3 days, then transferred to 22°C for an additional 3 days, stained with cotton blue and photographed. (B) Germ tube cell death. Spores were germinated at 22°C for 6 h, the germ tubes were transferred to 32°C for 24 h, stained with PI, and the percent of PI‐positive (dead) germ tubes was determined. Scale bars = 100 μm.

**Figure S3.** Mitochondria morphology without heat stress. Germ tubes were produced at 22°C for 24 h and then examined with a fluorescence microscope using a rhodamine filter (A) or TEM (B). M, mitochondria; N, nucleus; V, vacuole. Insets show mitochondria boundaries. Scale bars = 20 μm and 1 μm in A and B, respectively.

**Figure S4.** The red spots do not localize in mitochondria. Wild type and Δ*bclon1* spores were germinated on a cover slip at 29°C for 18 h. The samples were stained with the mitochondria MitoTracker green dye and images were captured using a fluorescent microscope with a GFP (for detection of MitoTracker) and Rhodamine (for detection of mCherry) filters. Scale bar = 10 μm.

**Figure S5.** Quantification of mitochondrial damage. Spores were germinated at 22°C or 29°C for 10 h, the germ tubes were collected and stained with Mito‐ID (for MMP) or DHR123 (for ROS). (A) Images of samples after staining with Mito‐ID. Top panel—22°C, bottom panel—29°C. Images were captured with a fluorescent microscope using rhodamine and GFP filters. Scale bars = 100 μm. (B) Relative levels of MMP. Fluorescence intensity was quantified using ImageJ and the ratio of red/green signal was calculated. (C) Images of samples after staining with DHR123. Images were captured with a fluorescent microscope using a YFP filter. Scale bars = 100 μm. (D) Relative levels of ROS. Fluorescence intensity was quantified using ImageJ. Graphs represent three (B, D) biological replications with overlaid individual data points. Values are presented as the mean of replicates ± SD. Statistical differences in all graphs were determined using unpaired two‐tailed Student’s *t*‐test (***p < 0.005).

**Figure S6.** Deletion of *bclon1* does not induce autophagy under optimal temperature. Germ tubes were produced at 22°C for 24 h, samples were stained with CMAC and images were captured with a fluorescent microscope using GFP and DAPI filters. Scale bars = 20 μm.

**Figure S7.** Effect of deletion of *bcatg1* and *bclon1* on spore germination and survival. (A, B) Spores were incubated at 22°C or 32°C and germination rates were determined after 3 or 8 h, respectively. (C, D) Seven‐day‐old cultures were incubated at 22°C (C) or 29°C (D), spores were collected every 7 days, stained with PI and the percentage of PI‐positive (dead) spores was determined. Graphs represent three (A, C, D) and four (B) biological replications with overlaid individual data points. Values are presented as the mean of replicates ± SD. Statistical differences were determined using unpaired two‐tailed Student’s *t*‐test (***p < 0.005).

**Figure S8.** Deletion of *bcatg4* and *bcatg9* blocks autophagy and affects spore morphology. (A) Monitoring of autophagy. Germ tubes were produced at 22°C for 6 h, the germ tubes were incubated for 18 h at 29°C, and then samples were stained with CMAC and inspected with a fluorescence microscope using the GFP and DAPI filters. Microscopic images of germ tubes were captured. Insets show induction (wild type) and blocking (∆*bcatg4* and ∆*bcatg9*) of autophagy. Scale bars = 20 μm. (B, C) Spore morphology and spore indexes. Fungi were cultured on PDA for 7 days, spores were collected, images were captured (B) and spore index I and II were calculated (C). Insets show abnormal spore morphology of autophagy mutant strains. Scale bars = 100 μm. (D) Spore germination. Spores were incubated at 32°C, and the germination rate was determined after 8 h. Graphs represent three (C, D) biological replications with overlaid individual data points. Values are presented as the mean of replicates ± SD. Statistical differences were determined using unpaired two‐tailed Student’s *t*‐test (*p < 0.05; **p < 0.01; ***p < 0.005).

**Table S1.** Fungal strains.

**Table S2.** List of plasmids.

**Table S3.** List of primers.

## Data Availability

The data that support the findings of this study are available in the Supporting Information of this article.
