# Supplementary figures and images for: Autophagy and the Mitochondrial Lon1 Protease Are Necessary for Botrytis cinerea Heat Adaptation
Source: Mol Microbiol. 2025 Jul 18;124(4):358–69. doi: 10.1111/mmi.70014 (PMC12510622; doi:10.1111/mmi.70014)

WT PDB

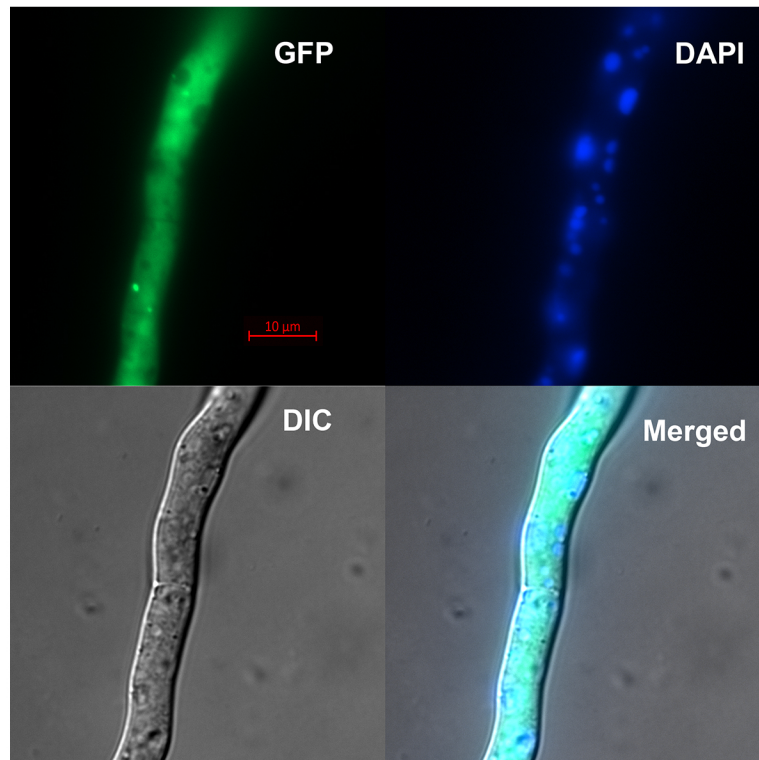

$\Delta bclon1$  PDB

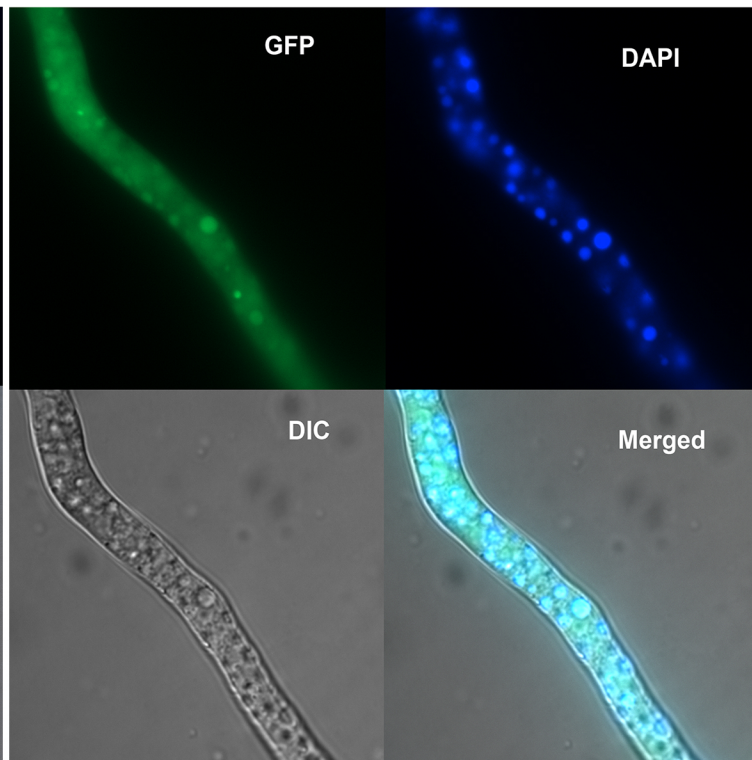

$\Delta bclon1$  MM

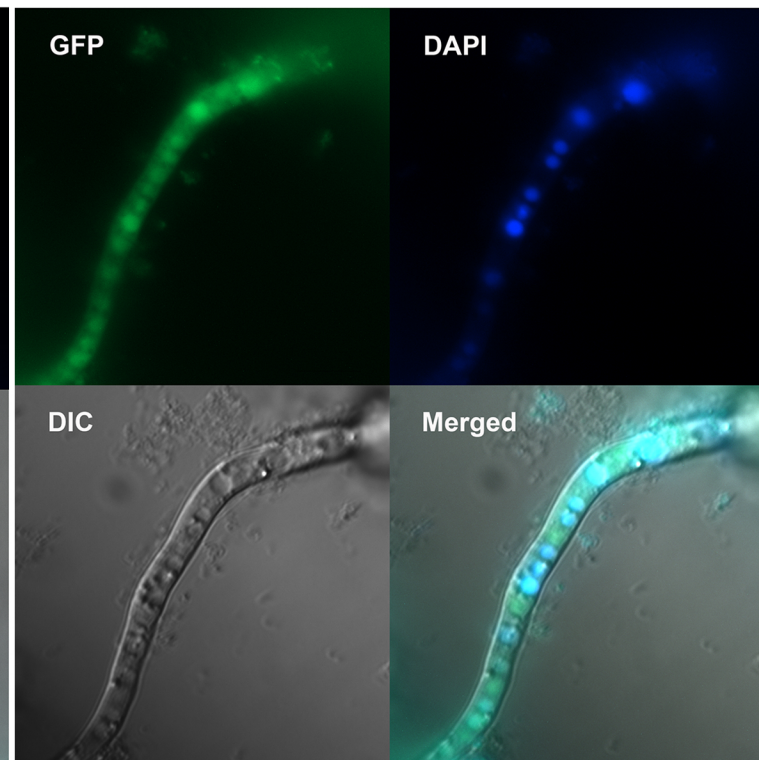

Supplement: Supplementary file 1 — Figure S1. Induction of autophagy by starvation. Spores were germinated at 22°C in PDB on coverslip for 6 h, then the PDB was replaced with fresh PDB (wt and Δbclon1 mutant) or with minimal medium (only Δbclon1 mutant) and the slides were incubated at 22°C for additional 18 h after which the hyphae were stained with CMAC. Images were captured using a fluorescent microscope with DAPI (for detection of CMAC staining) and GFP (for detection of ATG8‐GFP) filters. Scale bar = 10 μm. [file MMI-124-358-s006.pdf]

A

WT

 $\Delta bcatg1$  $\Delta bclon1$  $\Delta bcatg1/lon1$ 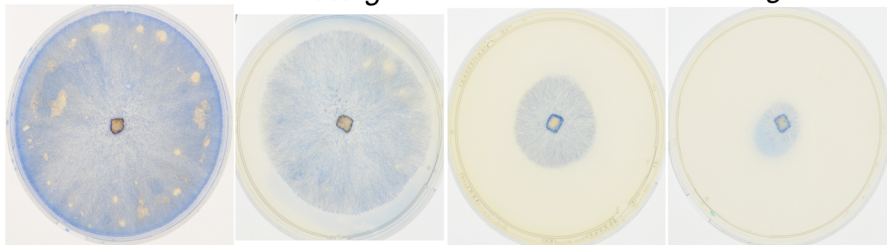

B

WT

 $\Delta bcatg1$  $\Delta bclon1$  $\Delta bcatg1/lon1$ 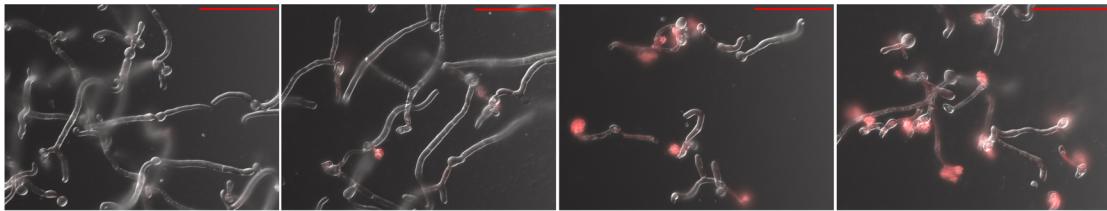

Supplement: Supplementary file 2 — Figure S2. Effect of autophagy and bclon1 on cell death and survival of mycelia and germ tubes. (A) Cultures were initiated on PDA from mycelial plugs. The plates were incubated at 32°C for 3 days, then transferred to 22°C for an additional 3 days, stained with cotton blue and photographed. (B) Germ tube cell death. Spores were germinated at 22°C for 6 h, the germ tubes were transferred to 32°C for 24 h, stained with PI, and the percent of PI‐positive (dead) germ tubes was determined. Scale bars = 100 μm. [file MMI-124-358-s005.pdf]

A

WT

 $\Delta bcatg1$  $\Delta bclon1$  $\Delta bcatg1/lon1$ 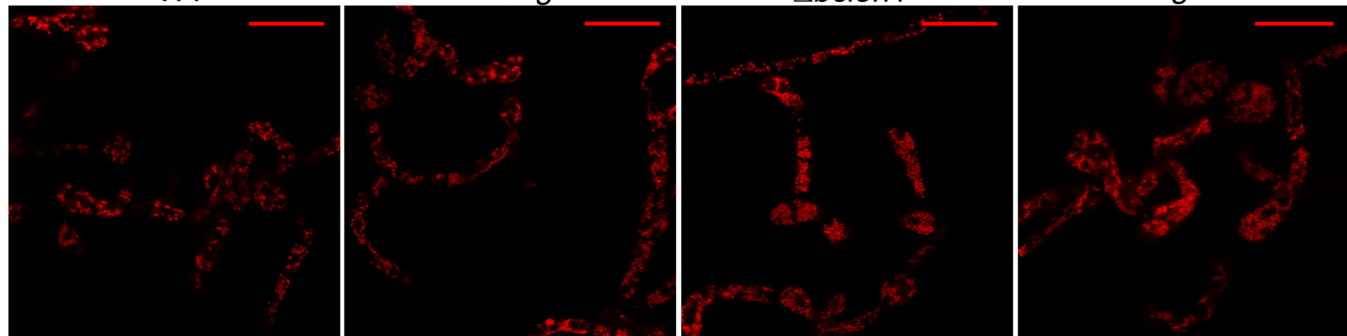

B

WT

 $\Delta bcatg1$  $\Delta bclon1$  $\Delta bcatg1/lon1$ 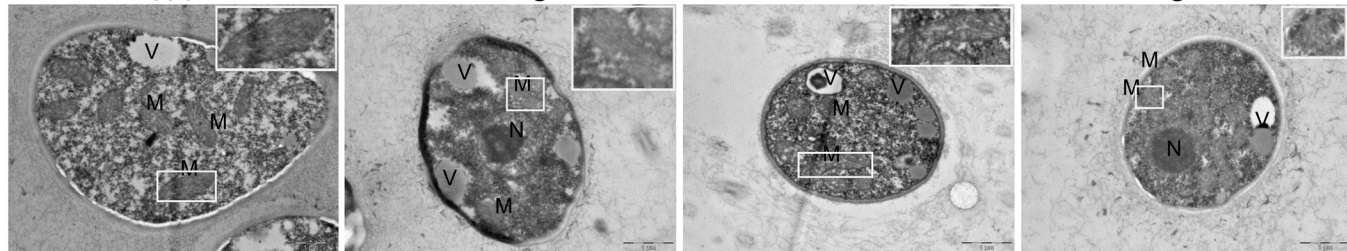

Supplement: Supplementary file 3 — Figure S3. Mitochondria morphology without heat stress. Germ tubes were produced at 22°C for 24 h and then examined with a fluorescence microscope using a rhodamine filter (A) or TEM (B). M, mitochondria; N, nucleus; V, vacuole. Insets show mitochondria boundaries. Scale bars = 20 μm and 1 μm in A and B, respectively. [file MMI-124-358-s009.pdf]

WT

$\Delta bclon1$

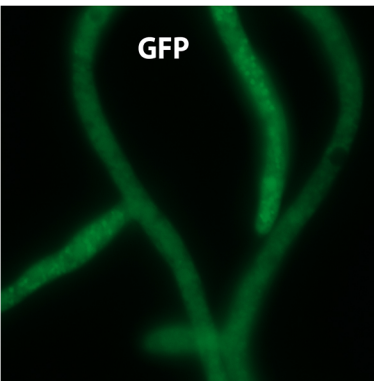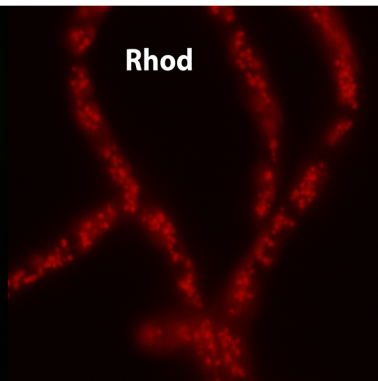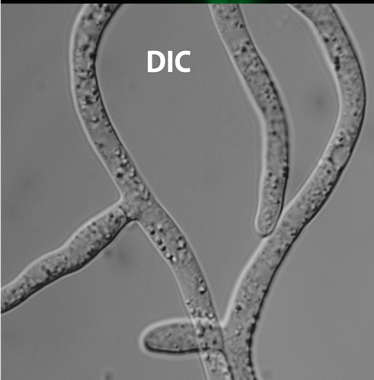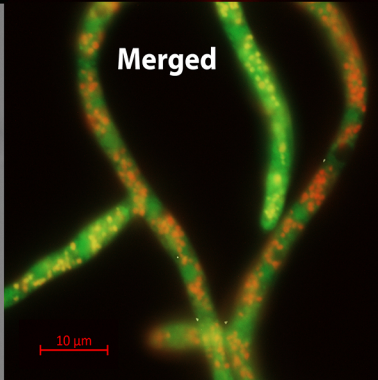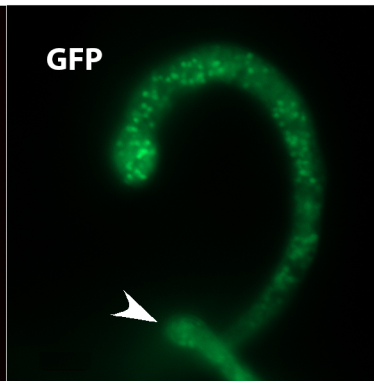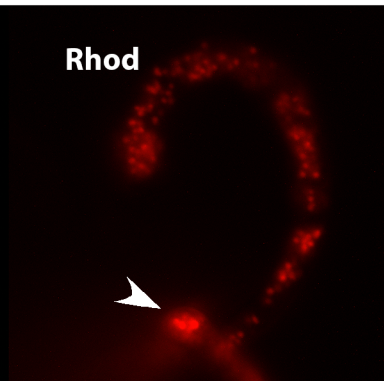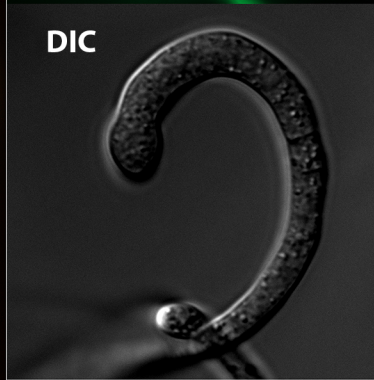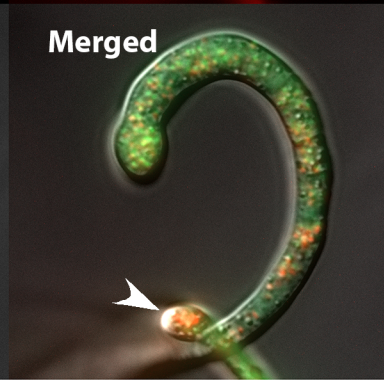

Supplement: Supplementary file 4 — Figure S4. The red spots do not localize in mitochondria. Wild type and Δbclon1 spores were germinated on a cover slip at 29°C for 18 h. The samples were stained with the mitochondria MitoTracker green dye and images were captured using a fluorescent microscope with a GFP (for detection of MitoTracker) and Rhodamine (for detection of mCherry) filters. Scale bar = 10 μm. [file MMI-124-358-s010.pdf]

**A**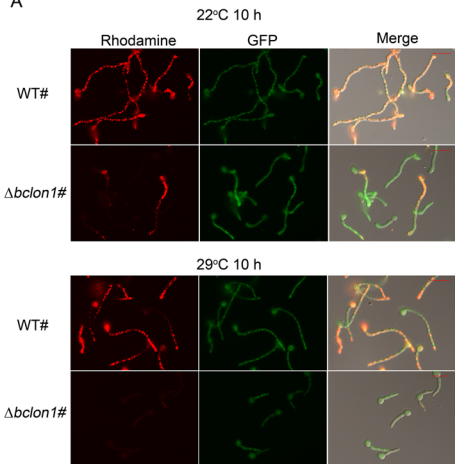**B**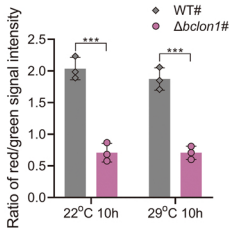**C**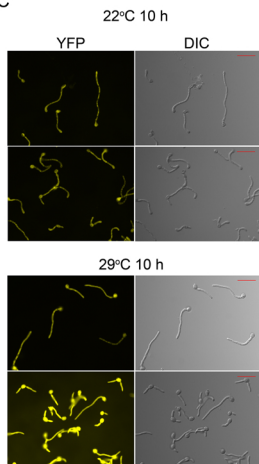**D**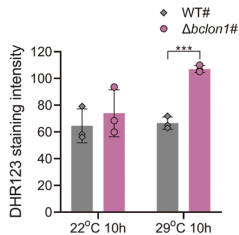

Supplement: Supplementary file 5 — Figure S5. Quantification of mitochondrial damage. Spores were germinated at 22°C or 29°C for 10 h, the germ tubes were collected and stained with Mito‐ID (for MMP) or DHR123 (for ROS). (A) Images of samples after staining with Mito‐ID. Top panel—22°C, bottom panel—29°C. Images were captured with a fluorescent microscope using rhodamine and GFP filters. Scale bars = 100 μm. (B) Relative levels of MMP. Fluorescence intensity was quantified using ImageJ and the ratio of red/green signal was calculated. (C) Images of samples after staining with DHR123. Images were captured with a fluorescent microscope using a YFP filter. Scale bars = 100 μm. (D) Relative levels of ROS. Fluorescence intensity was quantified using ImageJ. Graphs represent three (B, D) biological replications with overlaid individual data points. Values are presented as the mean of replicates ± SD. Statistical differences in all graphs were determined using unpaired two‐tailed Student’s t‐test (***p < 0.005). [file MMI-124-358-s002.pdf]

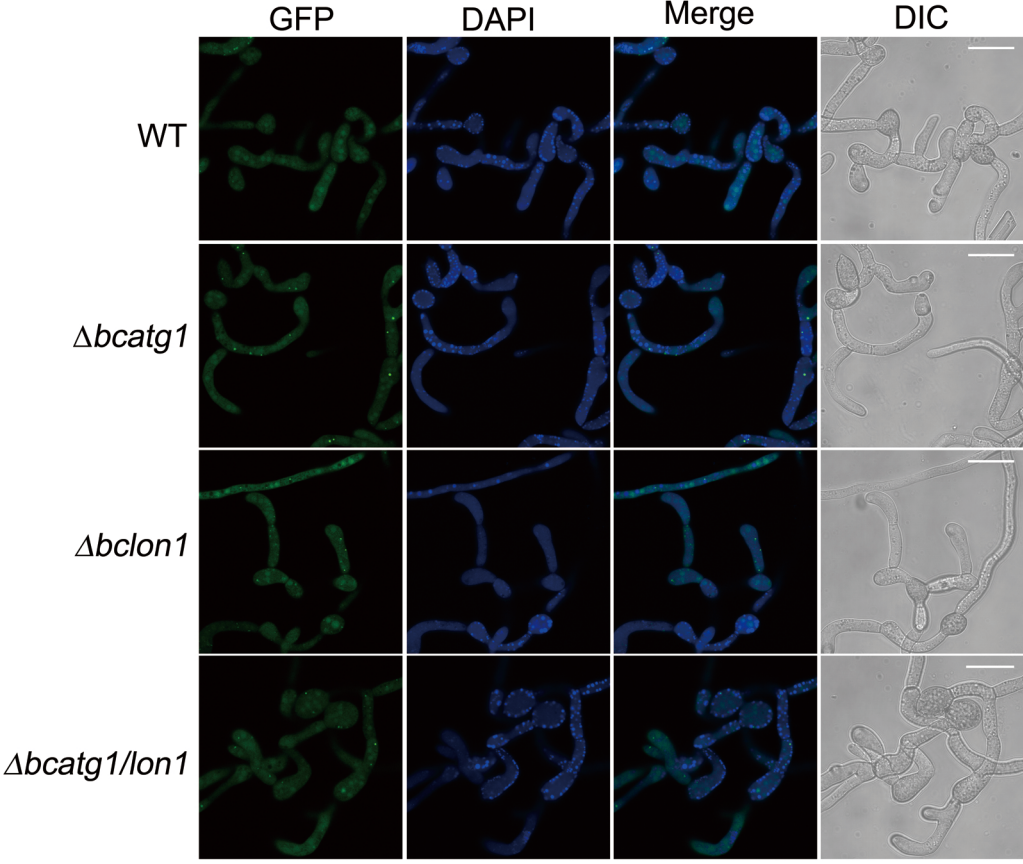

Supplement: Supplementary file 6 — Figure S6. Deletion of bclon1 does not induce autophagy under optimal temperature. Germ tubes were produced at 22°C for 24 h, samples were stained with CMAC and images were captured with a fluorescent microscope using GFP and DAPI filters. Scale bars = 20 μm. [file MMI-124-358-s011.pdf]

A

22°C

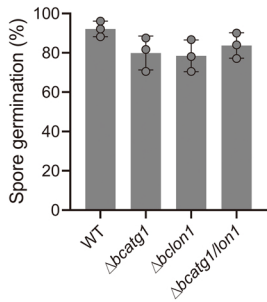

B

32°C

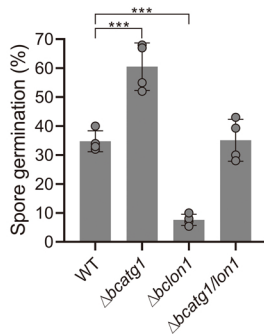

C

22°C

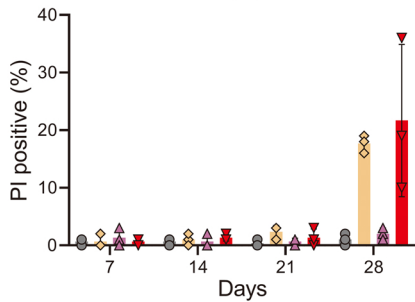

D

29°C

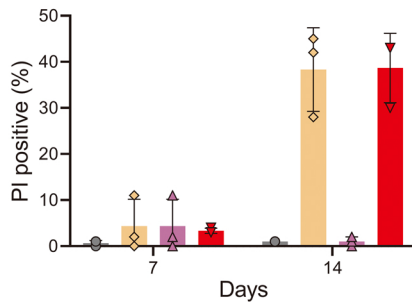

● WT    ◆  $\Delta bcatg1$   
 ▲  $\Delta bclon1$     ▼  $\Delta bcatg1/lon1$

Supplement: Supplementary file 7 — Figure S7. Effect of deletion of bcatg1 and bclon1 on spore germination and survival. (A, B) Spores were incubated at 22°C or 32°C and germination rates were determined after 3 or 8 h, respectively. (C, D) Seven‐day‐old cultures were incubated at 22°C (C) or 29°C (D), spores were collected every 7 days, stained with PI and the percentage of PI‐positive (dead) spores was determined. Graphs represent three (A, C, D) and four (B) biological replications with overlaid individual data points. Values are presented as the mean of replicates ± SD. Statistical differences were determined using unpaired two‐tailed Student’s t‐test (***p < 0.005). [file MMI-124-358-s007.pdf]

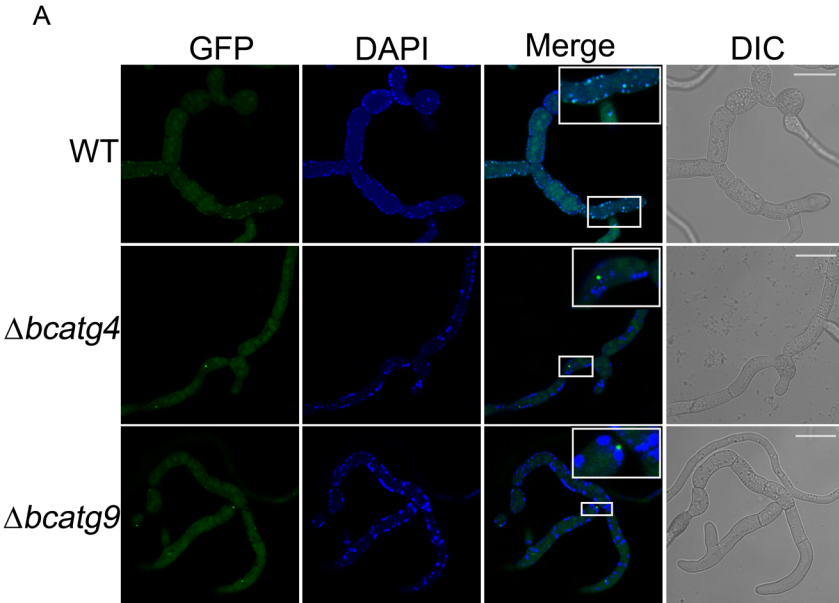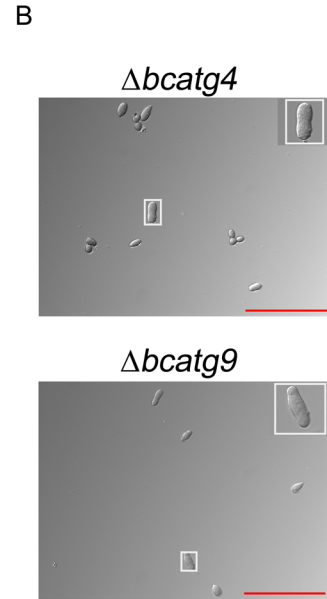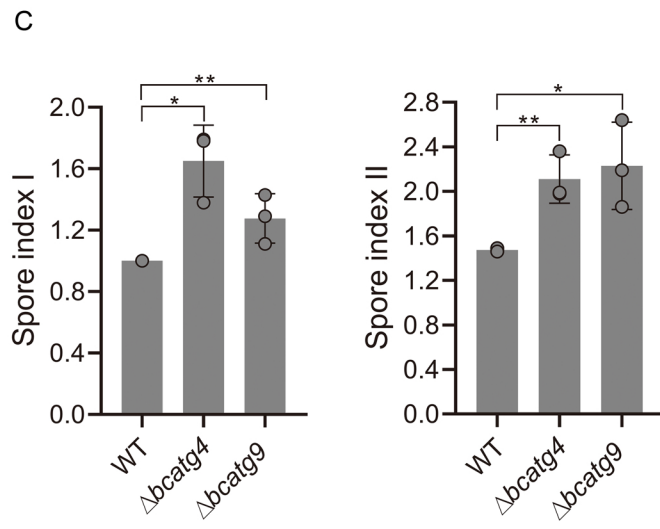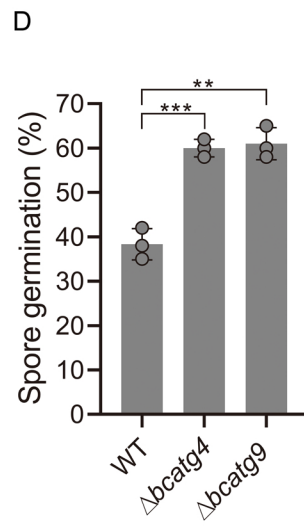

Supplement: Supplementary file 8 — Figure S8. Deletion of bcatg4 and bcatg9 blocks autophagy and affects spore morphology. (A) Monitoring of autophagy. Germ tubes were produced at 22°C for 6 h, the germ tubes were incubated for 18 h at 29°C, and then samples were stained with CMAC and inspected with a fluorescence microscope using the GFP and DAPI filters. Microscopic images of germ tubes were captured. Insets show induction (wild type) and blocking (∆bcatg4 and ∆bcatg9) of autophagy. Scale bars = 20 μm. (B, C) Spore morphology and spore indexes. Fungi were cultured on PDA for 7 days, spores were collected, images were captured (B) and spore index I and II were calculated (C). Insets show abnormal spore morphology of autophagy mutant strains. Scale bars = 100 μm. (D) Spore germination. Spores were incubated at 32°C, and the germination rate was determined after 8 h. Graphs represent three (C, D) biological replications with overlaid individual data points. Values are presented as the mean of replicates ± SD. Statistical differences were determined using unpaired two‐tailed Student’s t‐test (*p < 0.05; **p < 0.01; ***p < 0.005). [file MMI-124-358-s008.pdf]
