# Supplementary material for: Autophagy and the Mitochondrial Lon1 Protease Are Necessary for Botrytis cinerea Heat Adaptation
Source: Mol Microbiol. 2025 Jul 18;124(4):358–69. doi: 10.1111/mmi.70014 (PMC12510622; doi:10.1111/mmi.70014)
Supplement: Supplementary file 9 — Table S1. Fungal strains. [file MMI-124-358-s003.docx]

| **Strain** | **Description** | **Source** |
| --- | --- | --- |
| WT | A cassette to encode GFP-Atg8 and mt-mCherry was inserted at the 3' end of the *bcgpd* gene | This study |
| ∆*bcatg1* | A cassette to encode GFP-Atg8 and mt-mCherry was to replace the *bcatg1* gene | This study |
| ∆*bclong1* | A cassette to encode GFP-Atg8 and mt-mCherry was inserted at the 3' end of the *bcgpd* gene | This study |
| ∆*bcatg1/lon1* | A cassette to encode GFP-Atg8 and mt-mCherry was to replace the *bcatg1* gene | This study |
| ∆*bcatg4* | A cassette to encode GFP-Atg8 was to replace the *bcatg4* gene | This study |
| ∆*bcatg9* | A cassette to encode GFP-Atg8 was to replace the *bcatg9* gene | This study |
| WT# | *B. cinerea* strain B05.10 (For PI, Mito-ID and DHR123 staining) | Our lab |
| ∆*bcatg1*# | A Hygromycin resistance cassette was to replace the *bcatg1* gene of the WT# strain (For PI, Mito-ID and DHR123 staining) | This study |
| ∆*bclong1*# | A nourseothricin resistance cassette was to replace the *bclon1* gene (For PI, Mito-ID and DHR123 staining) | Our lab |
| ∆*bcatg1/lon1*# | A Hygromycin resistance cassette was to replace the *bcatg1* gene of the ∆*bclong1*# strain (For PI, Mito-ID and DHR123 staining) | This study |

**Table S1. Fungal strains**
