# Supplementary material for: Autophagy and the Mitochondrial Lon1 Protease Are Necessary for Botrytis cinerea Heat Adaptation
Source: Mol Microbiol. 2025 Jul 18;124(4):358–69. doi: 10.1111/mmi.70014 (PMC12510622; doi:10.1111/mmi.70014)
Supplement: Supplementary file 10 — Table S2. List of plasmids. [file MMI-124-358-s004.docx]

| **Plasmid** | | **Description** | | | | | | **Source** | | |  |  |  |  |  |
| --- | --- | --- | --- | --- | --- | --- | --- | --- | --- | --- | --- | --- | --- | --- | --- |
| POA-∆BCgpd-GFP-ATG8-Mt_mCherry | | *Bcgpdh* knockout vector, based on the pTZ57R/T plasmid. Carries Hygromycin B resistance cassette and mCherry under PH2B to label mithochondria and also GFP fused with ATG8, defined by the 5’ and 3’ flanking regions of *Bcgpd* gene for homologous recombination. | | | | | | This study | | |  |  |  |  |  |
| PTZ-∆ATG1-GFP-ATG8-Mt_mCherry | | *Bcatg1* knockout vector, based on the pTZ57R/T plasmid. Carries Hygromycin B resistance cassette and mCherry under PH2B to label mithochondria and also GFP fused with ATG8, defined by the 5’ and 3’ flanking regions of *Bcatg1* gene for homologous recombination. | | | | | | This study | | |  |  |  |  |  |
| PTZ-∆ATG4-GFP-ATG8 | | *Bcatg4* knockout vector, based on the pTZ57R/T plasmid. Carries Hygromycin B resistance cassette and GFP fused with ATG8, defined by the 5’ and 3’ flanking regions of  *Bcatg4* gene for homologous recombination. | | | | | | This study | | |  |  |  |  |  |
| PTZ-∆ATG9-GFP-ATG8 | | *Bcatg9* knockout vector, based on the pTZ57R/T plasmid. Carries Hygromycin B resistance cassette and GFP fused with ATG8, defined by the 5’ and 3’ flanking regions of  *Bcatg9* gene for homologous recombination. | | | | | | This study | | |  |  |  |  |  |
| PTZ-∆ATG1-Hyg | | *Bcatg1* knockout vector, based on the pTZ57R/T plasmid. Carries Hygromycin B resistance cassette, defined by the 5’ and 3’ flanking regions of  *Bcatg1* gene for homologous recombination. | | | | | | This study | | |  |  |  |  |  |
| PTZ-∆ATG1-GFP-ATG8 | | *Bcatg1* knockout vector, based on the pTZ57R/T plasmid. Carries Hygromycin B resistance cassette and GFP-ATG8 under PH2B to monitor autophagy, defined by the 5’ and 3’ flanking regions of  *Bcatg1* gene for homologous recombination. | | | | | | Our lab | | |  |  |  |  |  |
| PTZ-∆ATG1-Mt_mCherry | | *Bcatg1* knockout vector, based on the pTZ57R/T plasmid. Carries Hygromycin B resistance cassette and mCherry under PH2B to label mithochondria, defined by the 5’ and 3’ flanking regions of  *Bcatg1* gene for homologous recombination. | | | | | | Our lab | | |  |  |  |  |  |
| POA-∆BCgpd | | Vector based on the pTZ57R/T plasmid, defined by the 5’ and 3’ flanking regions of *Bcgpd* gene for homologous recombination. | | | | | | Our lab | | |  |  |  |  |  |
|  |  |  |  |  |  |  |  | |  |  | |  |  |  |  |

**Table S2. List of plasmids.**
